# Personalized *in vitro* Extracellular Matrix Models of Collagen VI-Related Muscular Dystrophies

**DOI:** 10.3389/fbioe.2022.851825

**Published:** 2022-04-25

**Authors:** Enrico Almici, Vanessa Chiappini, Arístides López-Márquez, Carmen Badosa, Blanca Blázquez, David Caballero, Joan Montero, Daniel Natera-de Benito, Andrés Nascimento, Mònica Roldán, Anna Lagunas, Cecilia Jiménez-Mallebrera, Josep Samitier

**Affiliations:** ^1^ Nanobioengineering Group, Institute for Bioengineering of Catalonia (IBEC), The Barcelona Institute of Science and Technology (BIST), Barcelona, Spain; ^2^ Department of Electronic and Biomedical Engineering, University of Barcelona, Barcelona, Spain; ^3^ Biomedical Research Networking, Center in Bioengineering, Biomaterials, and Nanomedicine (CIBER-BBN), Madrid, Spain; ^4^ Department of Mechanical and Aerospace Engineering, Politecnico di Torino, Torino, Italy; ^5^ Laboratorio de Investigación Aplicada en Enfermedades Neuromusculares, Institut de Recerca Sant Joan de Déu, Barcelona, Spain; ^6^ Unidad de Patología Neuromuscular, Servicio de Neuropediatría, Hospital Sant Joan de Déu, Barcelona, Spain; ^7^ Centro de Investigaciones Biomédicas en Red de Enfermedades Raras (CIBERER), Madrid, Spain; ^8^ Unitat de Microscòpia Confocal i Imatge Cel·lular, Servei de Medicina Genètica i Molecular, Institut Pediàtric de Malalties Rares (IPER), Hospital Sant Joan de Déu, Barcelona, Spain; ^9^ Department of Genetics, Microbiology and Statistics, University of Barcelona, Barselona, Spain

**Keywords:** extracellular matrix, Collagen VI related muscular dystrophy, *in vitro* model, decellularisation, patient-derived ECMs

## Abstract

Collagen VI-related dystrophies (COL6-RDs) are a group of rare congenital neuromuscular dystrophies that represent a continuum of overlapping clinical phenotypes that go from the milder Bethlem myopathy (BM) to the severe Ullrich congenital muscular dystrophy, for which there is no effective treatment. Mutations in one of the three Collagen VI genes alter the incorporation of this protein into the extracellular matrix (ECM), affecting the assembly and the structural integrity of the whole fibrillar network. Clinical hallmarks of COL6-RDs are secondary to the ECM disruption and include muscle weakness, proximal joint contractures, and distal hyperlaxity. Although some traits have been identified in patients’ ECMs, a correlation between the ECM features and the clinical phenotype has not been established, mainly due to the lack of predictive and reliable models of the pathology. Herein, we engineered a new personalized pre-clinical model of COL6-RDs using cell-derived matrices (CDMs) technology to better recapitulate the complexity of the native scenario. We found that CDMs from COL6-RD patients presented alterations in ECM structure and composition, showing a significantly decreased Collagen VI secretion, especially in the more severe phenotypes, and a decrease in Fibrillin-1 inclusion. Next, we examined the Collagen VI-mediated deposition of Fibronectin in the ECM, finding a higher alignment, length, width, and straightness than in patients with COL6-RDs. Overall, these results indicate that CDMs models are promising tools to explore the alterations that arise in the composition and fibrillar architecture due to mutations in Collagen VI genes, especially in early stages of matrix organization. Ultimately, CDMs derived from COL6-RD patients may become relevant pre-clinical models, which may help identifying novel biomarkers to be employed in the clinics and to investigate novel therapeutic targets and treatments.

## Introduction

Collagen type VI-related congenital muscular dystrophies (COL6-RD) constitute the second most frequent form of congenital muscular dystrophies (with a prevalence between 0.1 and 0.5 per 100,000 inhabitants), a group of neuromuscular diseases that cause degeneration in the muscular tissue (Graziano et al., 2015). They form a set of rare, disabling and yet incurable disorders whose origin and course are often unclear (Jimenez-Mallebrera et al., 2005). COL6-RD encompass a continuum of overlapping clinical conditions ranging from Bethlem myopathy (BM) to Ullrich congenital muscular dystrophy (UCMD), also with intermediate phenotypes (Lamandé and Bateman, 2018), (Natera-de Benito et al., 2021). Collagen VI (COL6) is a non-fibrillar component of the ECM expressed in many connective tissues and implicated in its organization (Cescon et al., 2015). Clinical hallmarks of COL6-RD are secondary to the ECM disruption and include muscle weakness, proximal joint contractures, and distal hyperlaxity (Bushby et al., 2014), (Zou et al., 2008). UCMD patients are at the severe end of the clinical spectrum: some of the affected children acquire the ability to walk independently but progression of the disease results in early loss of ambulation, need for nocturnal noninvasive ventilation (NIV) and decreased life expectancy (Lampe et al., 1993), (Ullrich, 1930). At the milder end of the clinical spectrum, patients with BM show a slower progression and even adult individuals often remain independently ambulatory (Natera-de Benito et al., 2021), (Jöbsis et al., 1996), (Hicks et al., 2008).

As described in the Online Mendelian Inheritance in Man (OMIM), COL6 structural defects are related to mutations in *COL6A1*, *COL6A2*, and *COL6A3* genes that encode for different chains, α1(VI), α2(VI) and α3(VI), which assemble in trimers to form the COL6 monomer (omim organization).^1,^ ^2^ These monomers further assemble into antiparallel dimers and then tetramers, stabilized by disulfide bonds, which are then secreted outside the cell. In the ECM, the tetramers associate in an end-to-end fashion giving rise to the final network with a characteristic beaded appearance (Cescon et al., 2015). However, the heterogenous set of mutations registered in patients modify the overall tetrameric structure of the protein by introducing alterations, often described as “kinks” or “wrinkles”, that are visible by electron microscopy (Butterfield et al., 2013). In general, pathologic COL6 fragments exert a negative effect on tetramers formed by non-mutated chains, altering their integration in the ECM and disrupting tissue homeostasis (Butterfield et al., 2013). COL6 interacts with multiple ECM key components, including fibrillar Collagens I and II, basement membrane Collagen IV, Fibronectin (FN), glycosaminoglycans and proteoglycans (Bonaldo et al., 1990; Fitch et al., 1991; Bidanset et al., 1992; Specks et al., 1992; Kuo et al., 1997; Guadagnin et al., 2021). Within the connective tissue, COL6 has been implicated in the modulation of ECM fibrillogenesis as well as in mediating the activity and availability of growth factors and cytokines (Ruehl et al., 2002), (Tillet et al., 1994).

Recent evidence has shown that primary fibroblasts cultures could be used to investigate the relationship between COL6-RD subtypes and ECM pathological signatures, and in particular, elastic fibers formation (Paco et al., 2015), (Allamand et al., 2011). Additionally, advanced image-based analyses of Collagen VI assembly may provide complementary information to routine diagnostic techniques (Bazaga et al., 2019). Therefore, it may be clinically relevant to investigate *in vitro* the structure of ECM networks to further understand the physiopathology of the disease and explore novel diagnostic and therapeutic strategies.

In this work, we describe the fabrication of a reductionistic pre-clinical model of the ECM by using cell-derived matrices (CDM) technology, as a first step towards the application of physiological models to COL6-RD. CDM consists of a structured bioengineering scaffold with *in vivo*-like features, which can function as an ECM model with disease-specific characteristics (Assunção et al., 2020). We used human dermal fibroblasts from COL6-RD patients and healthy donors to grow CDMs and study the structural properties of assembled ECM fibrils (Franco-Barraza et al., 2016). Overall, these models are promising tools to explore which alterations arise in the composition and fibrillar architecture due to patient mutations, especially in early stages of matrix organization.

## Results

### COL6-RD Donors and Controls Employed for CDM Preparation

Personalized CDMs were developed using forearm skin fibroblasts of five patients with COL6-RD and five healthy donors not affected by any neuromuscular condition as control. All patients had a confirmed diagnosis of COL6-RD based on genetic and/or immunohistochemical studies performed on muscle biopsy or dermal fibroblasts. The phenotype and genetic background of the patients are summarized in Table 1. Individuals had an average age of 7.2 years (range: 3-12) and harbored three different dominant mutations. Based on the phenotypic classification for COL6-RD, two individuals presented UCMD, two individuals intermediate COL6-RD, and one individual BM (Natera-de Benito et al., 2021).

**TABLE 1:** Control and COL6-RD donors included in the study. The table describes anonymously each fibroblast donor with details on the phenotype of the patients and the genetic background.

Alternative Name	Phenotype	Gene (Pattern of Inheritance)	Mutation	Age at Time of biopsy (years)	Sex
Patient 1	BM	COL6A1 (AD)	c.877G > A (Gly293Arg) Exon 10	9	Female
Patient 2	Intermediate	COL6A1 (AD)	c.877G > A (Gly293Arg) Exon 10	8	Male
Patient 3	Intermediate	—	Genetic confirmation pending	12	Female
Patient 4	UCMD	COL6A2 (AD)	c.901-2A > G Intron 7	3	Female
Patient 5	UCMD	COL6A1 (AD)	c.930 + 189C > T Intron 11	4	Male
Control 1	—	—	—	10	Male
Control 2	—	—	—	1	Male
Control 3	—	—	—	Exitus	Male
Control 4	—	—	—	2	Male
Control 5	—	—	—	3	Male

### CDMs Deposition

**FIGURE 1:**
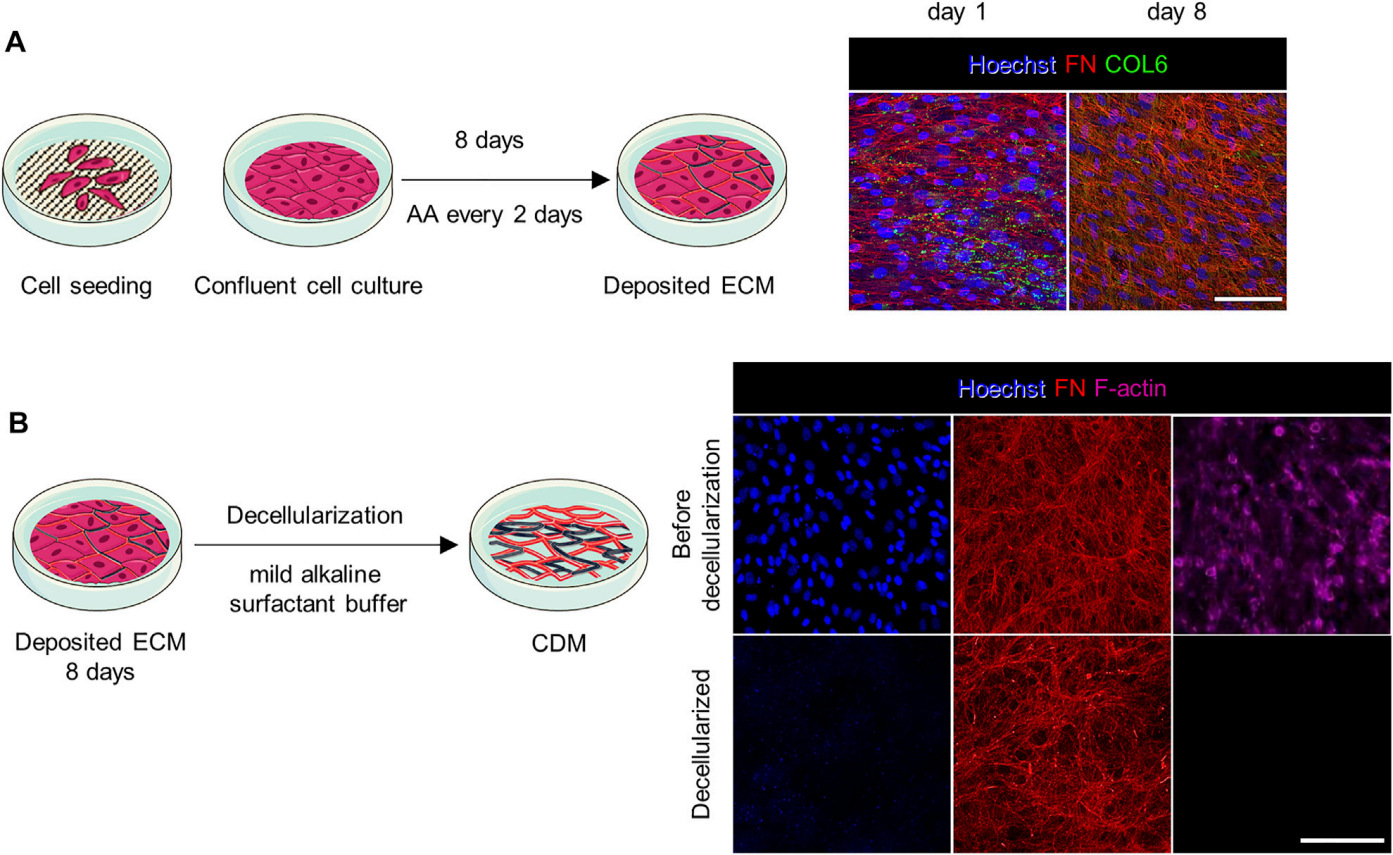
Preparation and application of CDMs. **(A)** Confluent cultures of fibroblasts were stimulated with AA for 8 days to promote the secretion of a dense fibrillar ECM rich in collagen. On the right, representative confocal images show cell staining at day 1 and 8 of culture (prior decellularization). Cell nuclei were stained with Hoechst. The complexity and interconnection of secreted fibrils increased over time. **(B)** The ECM was evaluated by immunostaining before and after decellularization. After decellularization no cellular structures such as cell nuclei (stained with Hoechst) and cytoskeleton (F-actin) were observed. Scale bars: 100 µm.

CDMs were produced adapting previous protocols (Franco-Barraza et al., 2016)– (Almici et al., 2020). Briefly, confluent cultures of fibroblasts from donors were stimulated to secrete and assemble collagen and FN-rich fibrillar networks over the course of multiple days. After 8 days of ascorbic acid (AA) stimulation, the cultures produced a dense fibrillar network, as highlighted by the FN and COL6 immunostaining (Figure 1A), which sustained the subsequent handling process. CDMs were obtained by decellularization using a mild alkaline surfactant buffer that disrupted the cells and helped to remove the debris; this treatment extracted the cellular components off the deposited ECM (Kaukonen et al., 2017). The resulting CDMs displayed a pristine network of fibrils (FN, Figure 1B) in total absence of any cellular structure (nuclei and F-actin, Figure 1B). Next, the CDMs were fixed and processed to investigate their architecture by immunofluorescence and image processing tools. The CDMs showed an heterogeneous fibrillar architecture and complex biochemical composition that provided to cells a native-like environment (Kaukonen et al., 2017), (Castelló-Cros et al., 2009), (Caballero et al., 2017).

### CDMs From Patients With COL6-RDs Show Deficiency in the Secreted COL6

**FIGURE 2:**
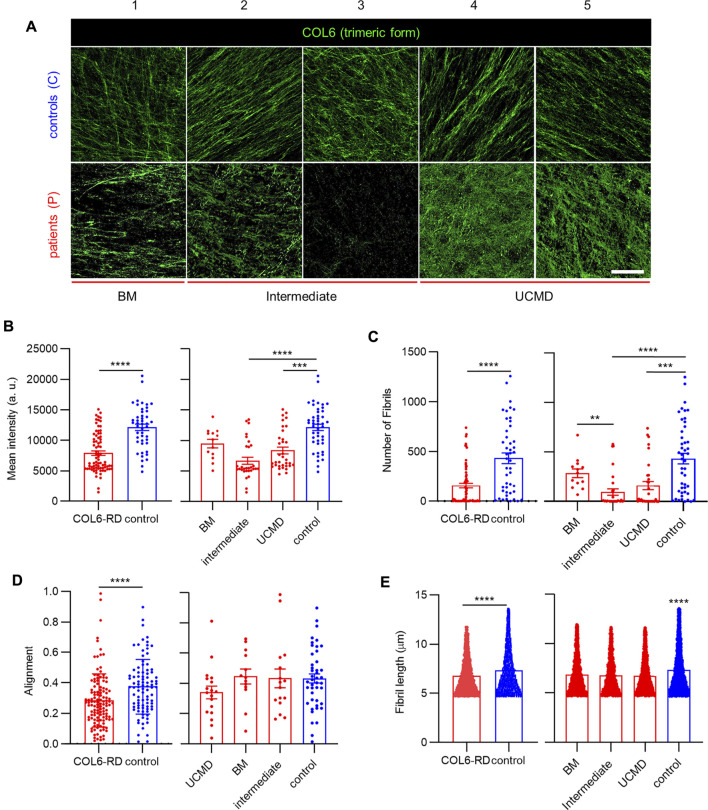
COL6 deposition in the CDMs. **(A)** Representative confocal images of COL6 in the CDMs obtained from patients with COL6-RDs and healthy donors (controls). Scale bar: 50 µm. **(B)** Quantification of the mean fluorescence intensity of COL6. 13 ≥ n ≥ 46, **(C)** Quantification of the number of fibrils. 13 ≥ n ≥ 46, **(D)** fibril alignment. 13 ≥ n ≥ 44, and **(E)** fibril length. 3413 ≥ n ≥ 18011, in CDMs through computational analysis, showing a significant decrease for patients with COL6-RDs. Three microscope fields of view were analyzed per replicate of at least 3 replicates per donor. Results are the mean ± SEM. **p < 0.01, ***p < 0.001, ****p < 0.0001. “a.u.”: arbitrary units.

To highlight the inclusion of structured COL6 in the fibrillar network of the ECM after decellularization, we stained for secreted COL6 in the CDMs. Previous studies indicated that the ensemble and secretion of the tetrameric form of COL6 is subjected to mutations, affecting microfibril organization in the ECM (Mercuri et al., 2002). Representative confocal images of the CDMs stained for COL6 are shown in Figure 2A and Supplementary Figure S1. Qualitatively, longer, and more defined microfibrils were found in control samples, which also presented a more homogeneous COL6 configuration among the different replicates. This was not the case for samples from patients with COL6-RDs, where shorter microfibrils were present in different amounts, and organized randomly. Patient 1 (P1), diagnosed with BM, exhibited the highest intensity of COL6 staining among patients. Patient 3 (P3), diagnosed with an intermediate phenotype as Patient 2 (P2) (Table 1), showed an almost total absence of COL6 staining in their CDMs. Interestingly, Patient 4 and 5, which were both diagnosed with UCMD, presented less drastic differences, confirming that the mutations encountered in patients impact COL6 secretion and assembly with different consequences for the ECM (Hicks et al., 2008), (Jimenez-Mallebrera et al., 2006). Quantification of the mean fluorescence intensity showed a significant reduction of COL6 staining in the CDMs of patients with COL6-RDs compared to the healthy donors. Intermediate and UCMD phenotypes contribute significantly to the differences observed, whereas the COL6 staining intensity in CDMs of patients with BM is similar to controls (Figure 2B). This result is in agreement with previous reports, showing that COL6 is often retained intracellularly and presents altered extracellular patterning in cultures of fibroblasts from patients (Hicks et al., 2008), (Jimenez-Mallebrera et al., 2006). Employing automatic fibrils reconstruction, we detected a significant difference in the number of fibrils, which are recognized in lower number in COL6-RD CDMs (Figure 2C), mirroring mean intensity results. We observed more aligned COL6 fibrils in control CDMs compared to patient’s counterpart (Figure 2D). Nonetheless, no difference could be highlighted between the different phenotypes. Fibril alignment calculation is based on circular descriptive statistics and consists in the mean resultant vector length that gives information on the spread of a circular variable in the population around its mean value (Berens, 2009), (Eliceiri et al., 2014). It describes the overall directionality of fibers within the image on a scale from 0 to 1, where 1 indicates that all fibers are orientated at the same angle. Finally, COL6 fibrils appeared significantly longer in controls compared to COL6-RD CDMs (Figure 2E). These results expand the qualitative description of COL6 alterations in patient fibroblast cultures suggested by Hicks *et al.* (Hicks et al., 2008)*.*

### Characterization of the Architecture of FN Fibrils in the CDMs

**FIGURE 3:**
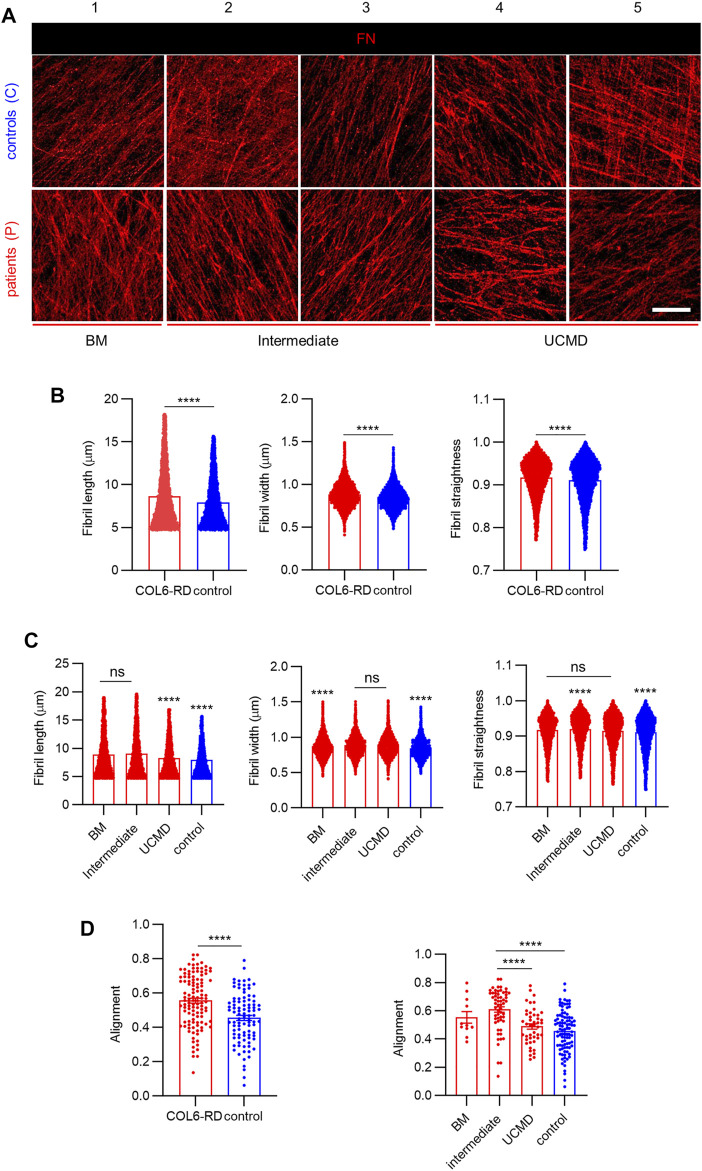
Analysis of FN fibrils distribution and morphology in the CDMs. **(A)** Representative images showing the immunostained FN fibrils obtained in the CDMs from healthy donors (controls) and from patients with COL6-RDs. Scale bar: 20 µm. **(B,C)** Quantification of FN fibril length. 4270 ≥ n ≥ 9042, width. 4815 ≥ n ≥ 9928, and straightness. 4654 ≥ n ≥ 9633, and **(D)** Quantification of fibrils alignment in CDMs. 11 ≥ n ≥ 94, through computational analysis. Two microscope fields of view were analyzed per replicate of at least 2 replicates per donor. Results are the mean ± SEM. ns = non-significant, ****p < 0.0001.

Previous results indicate that COL6 mediates the three-dimensional organization of FN in the ECM (Sabatelli et al., 2001), (Theocharidis et al., 2016). Therefore, we employed CDMs to further investigate alterations in the FN fibrillar architecture, which could be associated to mutations in *COL6* genes and COL6-RD patient phenotypes (Liu and Eliceiri, 2020), (Caballero and Samitier, 2017). Figure 3A shows representative images of FN immunostaining obtained from CDMs of patients with COL6-RDs and healthy donors (controls). The CDMs displayed fibrils of variable appearance showing interconnectivity and assorted orientation (Figure 3 and Supplementary Figure S2). Computational analysis was employed to quantify the structural properties of the CDMs. Similar to COL6, a significantly higher number of fibrils was found in control samples compared to those from patients with COL6-RDs (Supplementary Figure S3). However, on average, FN fibrils were found significantly longer, thicker, and straighter in CDMs derived from patients (Figure 3B). Fibril straightness was calculated as the ratio of the fibril length divided by its geodesic length, so that a perfectly straight fibril would have a value of 1 and increasing complexity (non-straightness) as the value approaches zero. Patients with distinct phenotypes showed differences among each other (Figure 3C), and intermediate phenotypes presented traits compatible with BM or UCMD outputs depending on the fibril characteristic that is being considered. FN fibrils were significantly shorter in patients with UCMD phenotype than those with intermediate and BM phenotypes, and fibrils were wider in CDMs of patients with BM, while no significant differences were found in the fibril width of CDMs from patients displaying intermediate and UCMD phenotypes. Also, the intermediate phenotype seemed to render straighter FN fibrils than BM and UCMD matrices.

Furthermore, we observed an increased alignment of FN fibrils in CDMs from COL6-RD patients (Figure 3D, left), which is in agreement with the results reported by Theocharidis *et al.* on COL6A1-depleted CDMs obtained from human dermal fibroblasts with stable knock-down of *COL6A1* (Theocharidis et al., 2016). In addition, we could observe in our patient-derived CDMs that the intermediate phenotype showed significantly more aligned FN fibrils compared to UCMD and control CDMs (Figure 3D, right).

### Inclusion of Elastic Fibrils in CDMs

**FIGURE 4:**
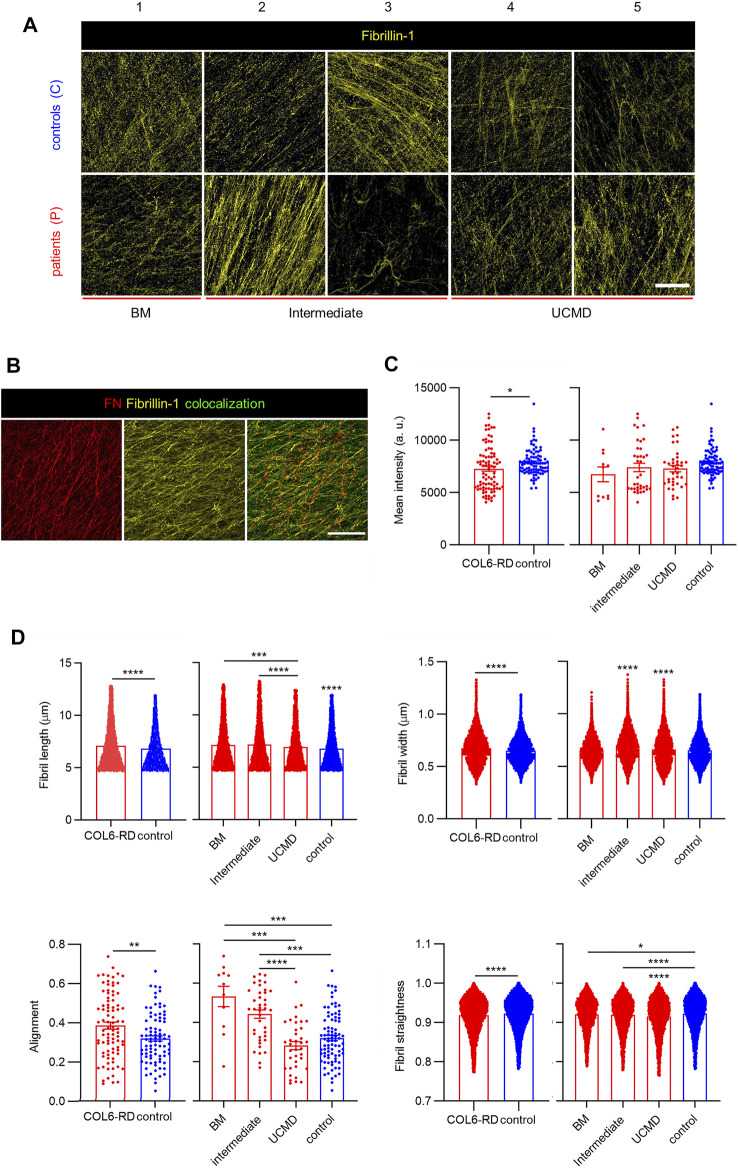
FBN1 deposition in CDMs. **(A)** Representative confocal images of FBN1 immunostaining in CDMs from healthy donors (controls) and patients with COL6-RDs. FBN1 immunolabelling show distinct motifs of secretion. **(B)** Representative images showing the co-localization of FBN1 with FN fibrils. Scale bars: 50 µm. **(C)** Quantification of the mean fluorescence intensity of FBN1 immunostaining. 11 ≥ n ≥ 86, showing its decrease for patients with COL6-RDs. **(D)** Quantification of FN fibril length. 4964 ≥ n ≥ 46439, width. 5497 ≥ n ≥ 50922, alignment. 11 ≥ n ≥ 87, and straightness. 5345 ≥ n ≥ 49168 in CDMs through computational analysis. Three microscope fields of view were analyzed per replicate of at least 3 replicates per donor. Results are the mean ± SEM. *p < 0.05, **p < 0.01, ***p < 0.001, ****p < 0.0001.

Finally, we investigated whether CDMs could be employed to study phenotypic changes in other ECM proteins linked to COL6 apart from FN. We focused on elastic fibers given their involvement in disease pathogenesis and cell-ECM regulation (Paco et al., 2015), (Thakkar et al., 2014). Fibrillins are ECM components, which assemble in microfibrils and serve as backbone for the assembly of elastic fibers, confer structural integrity and elasticity to tissues, and regulate growth factor signaling (Ramirez and Dietz, 2007), (Sabatier et al., 2009). Among them, and given its importance in the organization of elastin and its dependence on FN secretion, we investigated Fibrillin-1 (FBN1) (Thakkar et al., 2014), (Sabatier et al., 2009). Confocal images of FBN1 showed its presence in all the CDMs (Figure 4A). Even if the overall signal was weak, it can be appreciated that FBN1 was integrated within the ECM following different patterns, thereby confirming its involvement in ECM organization along with COL6 and FN. Similarly to the analysis of COL6, FBN1 levels for P3 in the immunostainings appeared much lower and leading to more disorganized fibers than for P2, although both patients have been diagnosed with an intermediate phenotype. Also from immunofluorescent images, we could observe the formation of FBN1 aggregates, complex fibrillar structures and fuzzy networks of fibrils that colocalized with FN (Figure 4B). Analysis of the mean fluorescence intensity of immunostained FBN1 in confocal projections showed that its inclusion in the CDMs was slightly lower in patients with COL6-RDs, although no significant differences could be found within the different phenotypes (Figure 4C). Accordingly, computational quantification of the number of FBN1 fibrils showed that they reproduced the analysis of the mean fluorescent intensity (Supplementary Figure S4). Like for FN, longer, thicker, and more aligned FBN1 arrangements were found in CDMs of patients with COL6-RDs. Nevertheless, in this case, control matrices presented straighter fibrils. Patients with different phenotypes show differences among each other. The intermediate phenotype behaves similarly to the BM phenotype for FBN1 fibril length, alignment, and straightness and more alike to UCMD phenotypes for the fibril width (Figure 4D).

## Discussion

### CDMs as Models of Pathological Changes in COL6-RD Patients’ ECM

In skin and muscle, two of the tissues affected in COL6-RD, fibroblasts are the main source of COL6 production, and therefore, one of the most relevant cell types involved in the pathogenesis of this disease (Jimenez-Mallebrera et al., 2006). Even if this cell type is in general recognized to be tissue-specific, processes that require their activation and function, such as fibrosis and wound healing, present similarities across connective tissues and their study can disclose key disease pathways given the central role of COL6 in ECM organization. For this reason, one of the key diagnostic tests to confirm or exclude a defect in COL6 in patients with suspected COL6-RD is to study the extent of reduction of COL6 *in vitro* cultures of fibroblasts derived from the patient´s skin (Fernández-Garibay et al., 2021). Therefore, skin fibroblasts can be exploited to derive CDMs that may reproduce key pathological features and biological variability of the patients *in vitro* (Paco et al., 2015), (Paco et al., 2013). Recent studies highlight the importance of the ECM and its interaction with cell receptors in the pathogenesis of COL6-RD (Paco et al., 2013). In this sense, CDM could be exploited to reproduce differences in composition and fibrils arrangement *in vitro*, which are characteristic of the tissue source and the genetic and pathologic background of patients (Chang et al., 2002), (Hoshiba and Tanaka, 2013). In this work, we show that CDMs obtained from skin fibroblasts of patients with COL6-RD present distinctive features compared to healthy donors, which recapitulate their underlying condition. Thereby, CDMs derived from patients with COL-RDs emerge as specific models representative of real pathological ECMs that can be employed to investigate disease traits to be used as complementary diagnostic tool, to study disease progression or for screening the efficacy of novel treatments. In this sense, CDMs are a simple yet informative *in vitro* model that can mimic patients’ ECM architecture and complexity (Franco-Barraza et al., 2016), (Ishikawa et al., 1997). Furthermore, CDMs have been previously shown to provide a suitable environment to inoculate secondary cultures of cells to investigate how disease-specific ECM signatures affect cells expression and response (Kaukonen et al., 2017), (Hoshiba and Tanaka, 2013). It is recognized that synthetic materials and matrices formed from isolated biological materials cannot achieve the molecular complexity and organization of native tissue ECM. CDMs obtained from skin fibroblasts are mostly representative of ECM secreted by fibroblasts during cutaneous wound repair, as it is deposited in the early stages of new tissue formation within the dermis (Theocharidis et al., 2016). Nonetheless, patients’ tissues affected by COL6-RDs are characterized by diminished COL6 as well as altered assembly and interaction with other ECM components (Jimenez-Mallebrera et al., 2006), (Paco et al., 2013). In particular, muscle biopsies were available for some of the patients included in this study and showed partial deficiency of COL6 in the basement membrane as shown in Paco *et al.* (Paco et al., 2013), (Paco et al., 2012). In this work, we presented for the first time the characterization of CDMs for primary COL6 deficiencies and there are still many open questions. Even if this is a first approximation, in any case it should not stop or undermine the investigation of 3D models which are more physiological than current standard system.

Compared to other bioengineering techniques that aim to recapitulate ECM characteristics, CDMs present less control on production and higher variability due to culture conditions, such as cell source, culture medium composition, initial culture substrates, and decellularization method (Hoshiba, 2017). Nevertheless, these constructs display a native-like composition and hierarchical ultrastructure which cannot be achieved with other *in vitro* techniques and biomaterials with complex composition, such as Matrigel™, which is from non-human origin (Kaukonen et al., 2017). Decellularized *ex vivo* tissues may be superior in recapitulating the complex ECM architecture, but offer low availability and present challenges in decellularization, reproducibility and ethical implications (Hoshiba and Tanaka, 2016). Finally, the development of complex tissues such as muscle and tendon *in vitro* is advancing at a fast pace (García-Lizarribar et al., 2018). In particular, new models for muscular dystrophies have been achieved with bioengineering techniques (Monferrer et al., 2020). These results manifest a promising future for the application of such tissues *in vitro*. These tools will enable the analysis of the biomechanics underlying patient symptoms and link it to neuromuscular diseases background in the future. Nonetheless, high throughput and patient-specific models are time-consuming and difficult to achieve with these techniques because they require multiple steps for the preparation of the culture supports and the establishment of cell cultures. Even if automatized and commercial systems are increasing in availability, there is still a significant amount of work to do to bring such advanced systems toward the clinical setting, first due to the multidisciplinary training necessary to exploit these tools, and next for the optimization steps that are still required. By contrast the CDM technology replicates very similar culture conditions and techniques already applied in COL6-RD histopathological assays, and may require only minimal specialized training (Jimenez-Mallebrera et al., 2006). Ultimately, even if more data is required to provide useful translational findings, CDMs appear as a promising tool to investigate and reproduce *in vitro* the biochemical signatures and biophysical properties of ECM from patients. Several evidence including the results presented here, highlight alterations in ECM composition and architecture depending on patient mutation and phenotypes. Therefore, it will be important in the future to consider these variables in modeling *in vitro* cell niches, with the aim of recapitulating relevant, patient-specific ECM properties. In this sense, CDM appear as a promising technique, compared to other conventional approaches, to provide personalized models that can recapitulate the complexity of ECM composition and structure with differences between normal and pathologic state.

### CDMs to Investigate COL6 Deficiency in ECM Composition and Architecture

To analyze the extent in which the identified mutations compromised the deposition of assembled COL6, we took advantage of the CDMs to evaluate the presence of trimeric COL6. We observed that its expression is significantly lower than in the CDMs of healthy donors, predominantly in intermediate and UCMD phenotypes. Especially in UCMD and intermediate COL6-RD fibroblasts cultures, mutations are reported to cause alterations to COL6 patterning, such as aggregates, reduced expression and secretion deficiency (Lamandé and Bateman, 2018), (Tagliavini et al., 2014), (Antoniel et al., 2020). Using automatic fibrils reconstruction, we found that less fibrils were deposited in the CDMs from patients with COL6-RDs and that they appeared shorter in length, and less aligned than those secreted in control CDMs. Shorter single microfilaments of COL6 have been reported to accumulate in the ECMs of proliferating tendon fibroblasts from patients displaying UCMD and BM phenotypes (Antoniel et al., 2020). COL6 interacts with several ECM proteins and it is implicated in collagens fibrillogenesis, fundamental for tissue assembly and function (Cescon et al., 2015), (Minamitani et al., 2004). Similarly, it has been previously reported the association of COL6 with FN, FBN1 and elastic fibers, underscoring its importance in controlling ECM organization (Zou et al., 2008), (Sabatelli et al., 2001), (Thakkar et al., 2014), (Kawahara et al., 2007). Exploiting CDM capacity to recapitulate key features of the native scenario, we investigated proteins implicated with COL6 in tissue development and structure (Thakkar et al., 2014). Previously, Theocharidis *et al.* downregulated *COL6A1* expression with shRNAs in immortalized cell lines, showing a strong impact in the architecture of the deposited FN network (Theocharidis et al., 2016). We observed that FN bundles length, thickness (higher in CDMs from patients with COL6-RDs) and disposition become altered with intermediate phenotypes presenting traits balanced within the extreme BM or UCMD phenotypes depending on the fibril characteristic considered (Figure 3). Our results showed increased FN alignment in CDMs from patients with COL6-RDs, in agreement with the characteristics reported by Theocharidis *et al.* on COL6-depleted CDMs, which presented aligned FN fibrils, more dispersed and thicker compared to controls (Theocharidis et al., 2016). Similarly, Sabatelli *et al.* and Antoniel *et al.* showed in cultured patients’ fibroblasts that deficiency of COL6 leads to aligned FN fibrils (Sabatelli et al., 2001), (Antoniel et al., 2020). Of note, our experiments were conducted employing primary fibroblasts that reflect the genetic background of patients and the most common scenario in COL6-RD, which is partially reduced COL6 rather than completely absent. Therefore, primary CDMs replicate the heterogeneity of real COL6-RD mutations and show variable extent of COL6 deficiency and related affectations in the ECM. Even if CDMs cannot achieve the exact molecular complexity and organization of native tissue ECM, this work paves the way for a broader characterization of COL6-RD patients’ ECM structure *in vitro* and for understanding the effect of different mutations. For example, our results suggest changes in FBN1 patterning in CDMs among patients’ phenotypes (Figure 4). The analysis of FN and/or FBN1 could be a secondary biomarker to confirm patients of suspected diagnosis when the deficit in COL6 may be of subtle interpretation. Future translational studies should be focused on increasing the size of the cohort of patients to refine and confirm this hypothesis, implementing image classification tools to avail a prompt clinical application (Bazaga et al., 2019). FBN1 labeling showed presence of aggregates, complex fibrillar structures and fuzzy networks of fibrils, moreover we detected a slightly lower immunofluorescence intensity in CDMs from patients with COL6-RDs (Figure 4). We observed colocalization between FBN1 and FN fibrils, as previously described and accounting for the role of FN in the organization of FBN1 in the ECM (Sabatier et al., 2009). Similarly to FN, the computational analysis of the structural properties of FBN1 fibrils showed higher length and width in CDMs from patients with COL6-RDs, and they were also more aligned. Its importance may be clinically and biologically relevant given the close association with FN and COL6 in connective tissues (Zou et al., 2008), (Sabatier et al., 2009), and the fact that microfibril assembly is commonly disturbed in patients affected by fibrillinopathies (presenting mutations in fibrillins genes) (Robinson et al., 2006).

In fact, we hypothesize that FBN1 could modulate ECM properties due to COL6 dysfunction, along with other proteins that have been shown to be expressed differently in patients (Paco et al., 2015), (Paco et al., 2013). In agreement with genomic and proteomic analyses, patients present characteristic signatures involving changes in ECM regulation that may be determinant in the development of their symptoms (Paco et al., 2015). Future refinement to this work may require to focus on assessing the interaction between multiple ECM components playing with inhibitors and genetic tools alongside with advanced image classification (Bazaga et al., 2019). Investigating the interplay of different ECM structures as well as cell signaling underlying fibrils deposition may contribute to answer key biological questions that have implication in COL6-RD. It is still not clear which is the mechanism underlying the joint contractures - most invalidating trait of the disease - and other connective tissue features of COL6-RD, but the cause is likely to be related to the association of COL6 with other ECM components and with receptors on the cell surface in those tissues (Paco et al., 2015), (Antoniel et al., 2020). Fibrillins are major components of elastic fibers, which are relevant in the context of COL6-RD and other connective tissue diseases where tendons and ligaments are also affected. The changes in FBN1 observed here in CDMs could result in alterations in the mechanical properties of the ECM, which may be probed directly on this model with atomic force microscopy (Otero et al., 2020). Nonetheless, these observations shall be confirmed in models recapitulating more closely the characteristics of musculoskeletal tissues. Future work may be developed exploiting other cell types (such as tenocytes or pluripotent stem cells), perhaps in association with organotypic dynamic *in vitro* culture to disclose how contractures develop in a patient- and mutation-specific manner (Testa et al., 2017), (Autonomous, 2018). Even in this context, CDMs may be employed to model with superior fidelity patients native ECM, compared to more conventional techniques to obtain 3D *in vitro* models.

## Conclusion

In this work, we obtained CDMs from COL6-RD patients and controls to characterize differences in their composition and architecture, partially depending on the severity of the phenotype. Although our study did not aim to investigate or disclose novel pathways in COL6-RD, we confirmed that patients’ skin fibroblasts present characteristic alterations in ECM protein secretion, and it is worth elucidating their impact on the disease pathogenesis. We could observe how the organization and compositions of CDMs appeared significantly different between healthy individuals and patients, suggesting a potential use of this tool in the future for more in-depth studies on COL6-RD *in vitro* models and ECM fibrils secretion. Furthermore, the application of CDMs along with advanced imaging and machine learning tools may improve the sensitivity with which alterations in patients’ ECM are observed and quantified, ultimately leading to improved diagnosis and monitoring of novel treatments (Bazaga et al., 2019). In conclusion, optimizing some of the drawbacks and completing the characterization of their complex composition, CDMs could be a powerful technique to address the need of patient-specific models for COL6-RD translational research in the near future.

### Ethical Aspects

The study has been approved by each entity by the respective Bioethics Commission (HSJD Ethics Committee and Bioethics Commission of University of Barcelona). Biological samples were obtained with written informed consent by the patients or their parents/guardians and custodied by the HSJD Biobank. This study has been performed according to current legislation on Biomedical Investigation in Spain, 14/2007 Law (BOE-A-2007-12945), the declaration of Helsinki (World Medical Asociation, 2013) and following good clinical and laboratory practices standards required by the European Research Commission.

## Methods

### Collection of Patients’ Fibroblasts.

Forearm skin biopsy were performed at Hospital Sant Joan de Déu, after written informed consent was obtained. Skin samples were collected from patients with COL6-RD and from children of the same age that were not affected by any neuromuscular condition. Briefly, skin samples were cut into small pieces, enzymatically digested to isolate the dermis, enriched with fibroblast cells. The sample was incubated in collagenase IV solution under shaking and centrifuged to obtain the fibroblasts, as previously described (Rittié and Fisher, 2005), (Henrot et al., 2020). In the fibroblasts culture, media was changed every 2-3 days for 3 weeks, by then the cells were confluent and therefore were frozen for storage (Jimenez-Mallebrera et al., 2006).

### Cell Culture

Patients’ forearm skin fibroblasts (Biobank from the Hospital Sant Joan de Déu) were grown in high-glucose Dulbecco’s Modified Eagle’s Medium (DMEM) (Invitrogen) supplemented with 1% Penicillin and Streptomycin (Invitrogen), 1% Sodium Pyruvate (Sigma-Aldrich) and 10% Fetal Bovine Serum (Sigma-Aldrich), at 37°C and 5% CO_2_. Cells were harvested at 80% confluence with 0.25% trypsin-EDTA (Life Technologies) for 5 min and employed until a maximum of 15 passages.

### CDM Deposition

The protocol for CDM growth was adapted from existing methods (Kaukonen et al., 2017), (Castelló-Cros and Cukierman, 2009). First, microscope coverslips (Ø 18 mm, Superior Marienfeld) were cleaned with soap and water, dried with N_2,_ and disinfected with ethanol and UV. Then, they were incubated with 1% sterile gelatin (from porcine skin, Sigma-Aldrich) in PBS and cross-linked with 1% filtered glutaraldehyde in PBS (Sigma-Aldrich). The reaction was quenched with 1 M sterile glycine (Sigma-Aldrich) solution in PBS. Gelatin coatings of a thickness of 1 ± 0.5 µm were obtained, for which an elastic modulus between 0.1-0.2 MPa has been reported, thus matching the elastic modulus range of human skin (Kuo et al., 2021), (Wei et al., 2017). The coated coverslips were used immediately or stored in the fridge with 1% Penicillin and Streptomycin in PBS for a maximum of two weeks. To assemble the matrices, 1 ml of medium containing 5 × 10^4^ cells/cm^2^ of primary fibroblasts were added on the gelatin-coated substrate. After one day, if confluency was achieved, ascorbic acid (AA) (Sigma-Aldrich) treatment was started. Standard culture media supplemented with 50 μg/ml AA was added every two days for a maximum of eight days to stimulate the generation of collagen and stabilize the generated matrix.

### Decellularization

On the eighth day of the AA treatment, the samples were decellularized: filtered extraction buffer, composed of 0.5% Triton X-100 (Sigma-Aldrich) and 300 mM of Ammonium hydroxide solution (NH_4_OH, Sigma-Aldrich) in PBS, was gently added on top of the samples, allowing cell lysis for 2 min, then it was diluted 5 times in PBS very gently and matrices stored overnight at 4°C. On the next day CDMs were rinsed carefully several times.

### CDM Immunostaining

The generated matrices were fixed right after decellularization with formalin 10% (Sigma-Aldrich) for 20 min. Then, samples were treated with a solution of ammonium chloride 50 mM in PBS (NH_4_Cl) (Sigma-Aldrich) to reduce the background due to aldehyde groups. Blocking and permeabilization was performed with a solution of 2% BSA and 0.2% Triton X-100 in PBS for 10 min at room temperature. Next, the matrices were stained overnight at 4°C with a combination of rabbit polyclonal anti-Fibronectin antibody (ab2413, Abcam) (1:200) and mouse monoclonal anti-Fibrillin-1 antibody (MAB2499 Millipore) (1:200), either mouse monoclonal anti-Collagen VI antibody (MAB3303, Millipore) (1:400) in PBS with 2% albumin from bovine serum (BSA, Sigma-Aldrich). On the next day, Alexa 568 goat anti-rabbit (A11036, Thermo Fisher Scientific) (1:1000) and Alexa 488 goat anti-mouse (A10667, Thermo Fisher Scientific) (1:1000) were used as a secondary antibody. The incubations were performed for 1 h at room temperature in PBS with 2% BSA. Samples were washed with PBS and mounted with Fluoromount (Sigma-Aldrich) on glass slides. Phalloidin−tetramethyl rhodamine B isothiocyanate (Sigma-Aldrich) was used for F-actin staining (1:1000). Nuclei were stained with Hoechst 33,342 (Molecular Probes) (1:1000). Both dyes were incubated for 1 h at room temperature.

### Confocal Microscopy

Zeiss LSM 800 (Carl Zeiss Microscopy GmbH, Jena, Germany) confocal laser scanning microscope (with 20x air or 40 × oil objectives, Plan-Apochromat of NA 0.8 and 1.4, respectively) was employed to acquire immunofluorescence images of 16 bits. The 3-dimensionality of the CDM was imaged with z-stacks acquired in increments of 0.5 μm. Excitation was performed with 488 and 561 nm diode lasers. Optimized emission detection bandwidths were configured at 400–570 nm (fluorochrome Alexa 488) and 570–700 nm (fluorochrome Alexa 568). For the experiments in Figure 2, excitation was performed with 405, 488 and 561 nm diode lasers and emission detection bandwidths were configured at 400-500 nm (Hoechst 33342), 500–560 nm (Alexa 488) and 570–700 nm (tetramethyl rhodamine B isothiocyanate). Power of the lasers and gain of the detectors were maintained constant across all samples.

### Analysis

Maximum intensity projections from confocal stacks were obtained and analyzed in Fiji and CurveletTransform—Fiber Extraction (CT-FIRE) algorithm (Schindelin et al., 2012), (Bredfeldt et al., 2014). Mean intensity and positive area of ECM immunostainings were calculated with Fiji. CT-FIRE algorithm was employed to reconstruct single FN fibrils and calculate the biophysical parameters straightness, length, width, alignment, and number of fibrils. Quantitative data was plotted as the mean with the standard error. Significant differences were judged using the One-way ANOVA with Tukey’s multiple comparisons test or *t*-test when only two groups are compared, using GraphPad Prism 8.0.2. When data did not pass a normality test, a Kruskal–Wallis test with Dunn’s multiple comparisons test or Mann Whitney test were applied instead. An *α* of less than 0.05 was considered statistically significant.

## Data Availability

The original contributions presented in the study are included in the article/Supplementary Material, further inquiries can be directed to the corresponding authors.

## Footnotes

1
https://omim.org/entry/158810

2
https://omim.org/entry/254090
