# Supplementary material for: Personalized in vitro Extracellular Matrix Models of Collagen VI-Related Muscular Dystrophies
Source: Front Bioeng Biotechnol. 2022 Apr 25;10:851825. doi: 10.3389/fbioe.2022.851825 (PMC9081367; doi:10.3389/fbioe.2022.851825)
Supplement: Supplementary file 1 [file DataSheet1.docx]

Supplementary Material

# Supplementary Figures


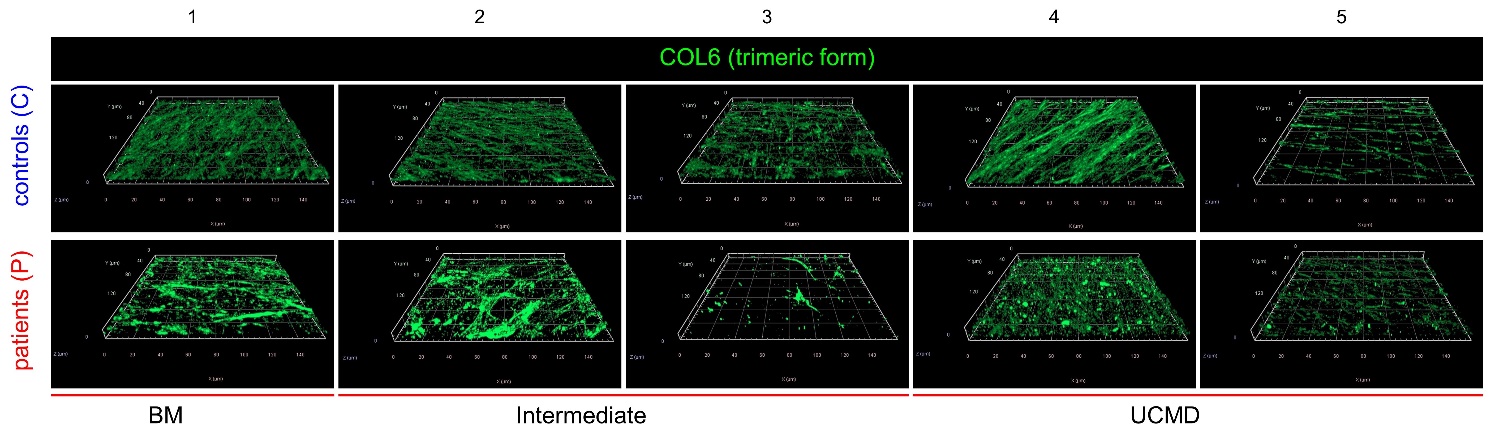


**Supplementary Figure 1.** Representative three-dimensional (3D) reconstruction of confocal images of COL6 in the cell-derived matrices (CDMs) obtained from patients with Collagen VI-related dystrophies (COL6-RDs) and healthy donors (controls). Three microscope fields of view were analyzed per replicate of at least 3 replicates per donor.

# Supplementary Figure 2. Rose plots of fibers angles from fibronectin (FN) immunostaining images reported in Figure 3.

1 2 3 4 5

patients (P)

controls (C)

UCMD

Intermediate

BM


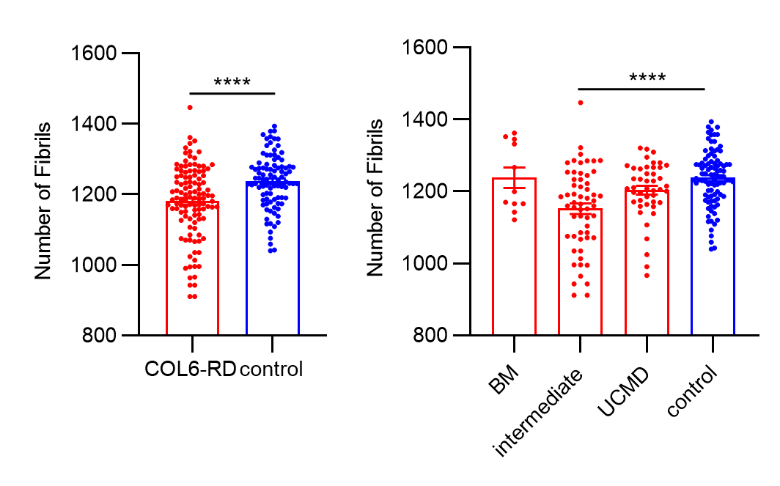


**Supplementary Figure 3.** Quantification of FN fibrils density, calculated as the number of fibrils in each image with CurveletTransform - Fiber Extraction (CT-FIRE). 11 ≥ n ≥ 94. Two microscope fields of view were analyzed per replicate of at least 2 replicates per donor. Results are the mean ± SEM. ****p < 0.0001.

**
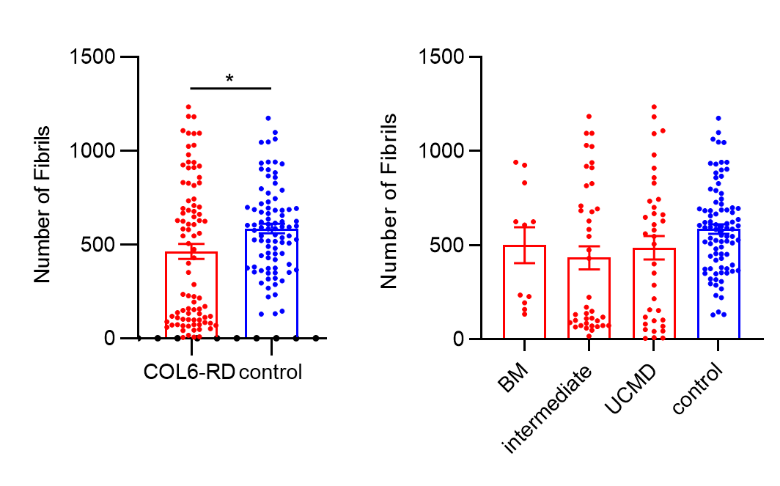
**

**Supplementary Figure 4** Quantification of fibrillin-1 (FBN1) fibrils density, calculated as the number of fibrils in each image with CT-FIRE. 11 ≥ n ≥ 87. Three microscope fields of view were analyzed per replicate of at least 3 replicates per donor. Results are the mean ± SEM. *p < 0.05
